# Opioid withdrawal delirium without convulsions: A Rare Case report

**DOI:** 10.1192/j.eurpsy.2022.2144

**Published:** 2022-09-01

**Authors:** D.S. Singh, S. Paul

**Affiliations:** 1 Neuro psychiatric and medical surgical centre, Psychiatry, pathankot, India; 2 Neuro psychiatric and medical surgical centre, Anaesthesiology, pathankot, India

**Keywords:** siezures, rare case report, opiod, addiction, delerium, convulsions, opiod add addiction add delerium add mconvulsions

## Abstract

**Introduction:**

Opioid withdrawal symptoms classically include severe muscle cramps, bone aches, autonomic symptoms, anxiety. Patients seldom have other complications like delirium and convulsions unless they have comorbid medical illnesses.

**Objectives:**

We hereby report a case of opioid withdrawal delirium.

**Methods:**

A 20-year-old man with dependence for opiods and nicotine was admitted after compete history and mental status and physical examination, last intake for both substances 2 days back. There was no history of fever, head injury, siezures and other substance use. All investigations done were normal and urine drug screen was negative for other substances. Treatment was started with clonidine and quetiapine for sleep and Nsaids on prn basis. After 2 days there was hallucinatory behaviour, agitation, fleeting episodes of recognising family members, hearing voices and decreased sleep observed. Patient required sedation with 10 mg of lorazepam and haloperidol before he went to sleep.Later on lorazepam 8 mg in divided doses and clonidine was tapered off gradually and patient as discharged on naltrexone 50mg.

**Results:**

In our case we could not find any other reason for delirium.These complications are rare feature of delirium, parker et all reported 5 such cases. One of limitations was we didnt do blood alcohol levels which could have ruled out alcohol use.

**Conclusions:**

This case is unique in terms of presenting with delirium without convulsions after 4 days of abstinence. No associated comorbidities, organic causes, and other substance use in dependence pattern or recently used. Use of a street variety (mixed with impurities) could be a risk factor for delirium in our patient.Psychiatrist need to be aware of complication.

**Disclosure:**

No significant relationships.

